# The wilt pathogen induces different variations of root-associated microbiomes of plant

**DOI:** 10.3389/fpls.2022.1023837

**Published:** 2022-09-16

**Authors:** Jiemeng Tao, Shizhou Yu, Jingjing Jin, Peng Lu, Zhixiao Yang, Yalong Xu, Qiansi Chen, Zefeng Li, Peijian Cao

**Affiliations:** ^1^ China Tobacco Gene Research Center, Zhengzhou Tobacco Research Institute of CNTC, Zhengzhou, China; ^2^ Molecular Genetics Key Laboratory of China Tobacco, Guizhou Academy of Tobacco, Guiyang, China

**Keywords:** root-associated microbiomes, bacterial wilt disease, disease suppression, microbiome assembly, microbial networks

## Abstract

Root-associated compartments, including the rhizosphere, rhizoplane, and endosphere, live with diverse microbial communities which profoundly affect plant growth and health. However, a systematic understanding of the microbiome assembly across the rhizosphere, rhizoplane, and endosphere under pathogen invasion remains elusive. Using 16S high-throughput sequencing, we studied how bacterial wilt disease affected the variation and assembly of the three continuous root-associated microbiomes of tobacco. The results indicated that microorganisms were gradually filtered from the rhizosphere to the endosphere. With the pathogen invasion, the rhizosphere, rhizoplane and endosphere microbiomes selected and recruited different beneficial bacterial taxa. Some recruited bacteria were also identified as keystone members in networks (i.e., *Bosea* in the endosphere). The microbiomes of endosphere and rhizoplane were more sensitive to plant disease than the rhizosphere microbiome. Still, response strategies of the rhizoplane and endosphere to disease were obviously different. Microbial networks of the rhizoplane became complex in diseased samples and genes involved in sporulation formation and cell cycle were enriched. However, microbial networks of the diseased endosphere were disrupted, and functional genes related to nitrogen utilization and chemotaxis were significantly increased, indicating the importance of nitrogen resources supply of plants for the endosphere microbiome under pathogen invasion. Our results provide novel insights for understanding the different responses of the root-associated microbiomes to plant disease.

## Introduction

In nature, plants do not grow as solitary organisms but harbor a high diversity of microorganisms. These microorganisms, which inhabit the rhizosphere, rhizoplane, phyllosphere, and endosphere, form microbial communities and are collectively called plant microbiomes (Turner et al., 2013; Müller et al., 2016). The plant microbiomes coevolve with their host and profoundly impact a range of aspects of plant performance (Simon et al., 2019; Trivedi et al., 2020), including nutrient availability (Martin et al., 2017), abiotic stress tolerance (de Vries et al., 2020), and disease suppression (Mendes et al., 2011; Carrión et al., 2019). Therefore, understanding the plant microbiomes will have important implications for plant health, productivity, and ecosystem function (Sessitsch and Mitter, 2015).

Soil is the largest known reservoir of microbial diversity (Torsvik et al., 2002), and the root is a critical zone for plant-microbiome interactions. The root zone can be separated into three rhizocompartments: the rhizosphere (soil close to the root surface), rhizoplane (root surface), and endosphere (root interior), each of which was found to harbor a distinct microbiome (Edwards et al., 2015). Recently, a three-step enrichment model was proposed to demonstrate the forming process of the three compartments at the soil-root interface (Reinhold-Hurek et al., 2015). The suitable physicochemical environment and rich nutrient conditions of the roots can recruit microorganisms migrating from the bulk soil to the roots. In this process, a portion of microorganisms is enriched in the soil close to the roots and forms the rhizosphere microbiome. Then, a specialized community is further enriched on the root surface and forms the rhizoplane microbiome. Finally, some microorganisms which have higher compatibility with plants enter and occupy niches within plant roots and establish the endosphere microbiome to greatly influence plant growth and health (Reinhold-Hurek et al., 2015). It is believed that the microbial communities inhabiting the three rhizocompartments are mainly derived from the bulk soil and gradually filtered at different compartment niches (Xiong et al., 2021). However, whether the enrichment process of the three root-associated microbiomes is affected by biotic stresses such as disease remains elusive.

Previous studies have suggested that changes in plant microbiomes are not just passive responses of plants but rather the result of coevolution, and plants may actively seek help from microbes to relieve stresses (Liu et al., 2020). The recruitment of microbes upon biotic or abiotic stresses is likely a survival strategy conserved across the plant kingdom, known as the “cry for help” strategy (Gao et al., 2021; Liu et al., 2021). Plants can recruit some beneficial microbes from surrounding soils, and the structure and function of plant microbiomes change and then reassemble (Xiong et al., 2021). For example, *Stenotrophomonas rhizophila* (SR80) was enriched in both the rhizosphere and root endosphere of *Fp*-infected wheat, and re-inoculation of SR80 in soils could enhance both plant health and disease suppression (Liu et al., 2021). Fusarium wilt disease greatly influenced the bacterial and fungal community assembly of the root and stem microbiomes and potentially beneficial bacteria such as *Pseudomonas*, *Streptomyces*, and *Bacillus* were enriched in diseased pepper plants (Gao et al., 2021). Microbial community assembly is mainly influenced by the host, environmental factors, and potential interactions among microbial individuals (van der Heijden and Hartmann, 2016; Durán et al., 2018). The cooperative and competitive interactions among different microbes across different habitats can be characterized using microbial co-occurrence network analysis (Feng et al., 2019). A growing number of studies have provided evidence that keystone members (e.g., connectors, module hubs, and network hubs) in microbial networks are crucial for predicting plant growth and fitness (Tao et al., 2017; Hu et al., 2020; Tao et al., 2021). However, most previous studies often focused on the rhizosphere microbiome, and a systematic understanding of the microbiome assembly across the rhizosphere, rhizoplane, and endosphere under pathogen invasion remains unclear.

Bacterial wilt disease is caused by *Ralstonia* species which infect a wide variety of *Solanaceae* crops, such as tobacco, tomato, and eggplant (Jiang et al., 2017). The *Ralstonia* pathogen survives in soils, enters the plant through root wounds, and destroys the vascular bundle system of the host, leading to the wilting and death of the host plant (Li et al., 2017). To reveal the structure, variation, and assembly of the three continuous root-associated microbiomes with *Ralstonia* invasion, soil and root samples were collected from healthy or diseased fields with tobacco plants. Using 16S rRNA gene sequencing, we aim to (i) describe the taxonomic and functional shifts in the three root-associated microbiomes under bacterial wilt disease, (ii) investigate which members in different microbiomes conferred positive effects on the plant upon pathogen attack, and (iii) compare the networks of healthy and diseased root-associated microbiomes to offer insights into the stability of communities.

## Materials and methods

### Sample collection and processing

All samples were collected from the main tobacco-growing regions in Tianzhu (26°58’15’’ N, 109°15’34’’ E), Guizhou province in China. The annual mean temperature in Tianzhu is 16.1°C, and the average annual precipitation was between 1200 mm to 1380 mm. The tobacco cultivar planted was Yunyan 87, and sample collections were performed on 4th September 2021. There were two adjacent plots in which tobacco plants displayed considerably different wilt symptoms. Tobaccos in the healthy field showed healthy conditions, and those in the diseased field showed severe wilt symptoms (infection grade 5–9). Six replicates of both soils and roots were taken from healthy and diseased fields, respectively. Each replicate was a composite sample formed by mixing two individual samples.

The bulk soil was collected 20 cm away from the root at 0–15 cm depth. The three rhizocompartments were gradually collected according to previous studies with some modifications. The whole plant was uprooted from the soil. The loosely attached soil was manually shaken from the roots, and the rhizosphere soil was collected by gently brushing the remaining soil on the roots (Zhang et al., 2017). The roots with the rhizosphere compartment removed were placed in a 50 mL tube with 15 mL of sterile Phosphate Buffered Saline (PBS) solution. After sonicating at 40 kHz for 1 min, the PBS was transferred into a new clean tube. Refilled PBS in the sonicated roots and repeated the same sonication procedure until no cells in the supernatant could be detected by microscopy. The mixed PBS solution was centrifuged at 12,000 × g for 10 min to collect rhizoplane microbes (Edwards et al., 2015). The sonicated roots were stored at -80°C for endosphere DNA extraction.

### DNA extraction and amplicon sequencing

Each plant sample was divided into four compartments: the bulk soil, the rhizosphere soil, the rhizoplane, and the endosphere. A total of 0.5 g bulk soil, rhizosphere soil, or rhizoplane microbial enrichment were used to extract DNA using a Mag-Bind^®^ Soil DNA Kit (Omega Biotek Inc., Doraville, GA, USA) following the manufacturer’s instructions. For the root endosphere, about 5 g sonicated sample as above was surface sterilized by consecutive immersion for 5 min in 75% ethanol, 5 min in 1% sodium hypochlorite solution, and 30 s in 75% ethanol and finally washed with sterile H_2_O for three times. The sterilized roots were ground using sterile mortars and pestles with liquid nitrogen, and then DNA was extracted from the 0.4 g resulting powder using the Mag-Bind^®^ Soil DNA Kit. The DNA concentration was evaluated using a NanoDrop^®^ 1000 spectrophotometer (Thermo Scientific), and the DNA quality was assessed by 1.0% agarose gel. The V3-V4 region (341F/805R) and V5-V7 region (799F/1193R) of bacterial 16S rRNA gene were amplified for epiphytic and endophytic microbes, respectively. Primer sequences and PCR amplification conditions are shown in **Table S1**. PCR was conducted in 25 μL mixtures containing 12.5 μL TaqMaster Mix (Vazyme, Piscataway, NJ, USA), 2.5 μL of forward/reverse primers (1 μM), 2.0 μL DNA (~25 ng/μL), and DNase-RNase-Free deionized water to adjust the volume. PCR products were purified using the E.Z.N.A. TM Gel Extraction Kit (OMEGA Biotek Inc., Doraville, GA, USA). Purified PCR products were pooled in equimolar concentrations for library construction. High-throughput sequencing was performed on the MiSeq platform (Illumina, San Diego, CA, USA).

### Amplicon sequencing data processing

The 16S rRNA gene sequences were processed using QIIME 2 (Quantitative Insights Into Microbial Ecology 2, version 2019.7) (Bolyen et al., 2019) and USEARCH v10.0 (Edgar, 2010). DATA2 (Callahan et al., 2016) was used for quality control, chimeric filtering, and to infer unique sample sequences (amplicon sequence variants, ASVs) from the sequencing data. The ASVs were subsequently assigned to taxonomy groups by comparisons with the SILVA v138 prokaryotic database (Yilmaz et al., 2014). Alpha diversity indices (Shannon, Chao1, and Simpson) and beta diversity metrics (Bray-Curtis) of the bacterial community were calculated in QIIME 2 through the diversity plugin (q2-diversity). To eliminate the influences of different sequencing depths, the bacterial ASV tables were rarefied to 29,546 reads for alpha diversity index estimates. The Phylogenetic Investigation of Communities by Reconstruction of Unobserved States (PICRUSt2) was applied to predict potential functional profiles of the bacterial community using 16S rRNA gene data (Douglas et al., 2020).

### Statistical analysis

All statistical analyses were performed using specific packages in R version 3.6.3 (The R Foundation for Statistical Computing, Vienna, Austria). Alpha diversity indices (Shannon index, Chao1 index, and Simpson index) of the bacterial community were calculated in QIIME 2. The beta diversity of bacterial communities was assessed by computing Bray-Curtis distance matrices and visualized using Principal coordinate analysis (PCoA) plots. The relative contribution of different factors to community dissimilarity was evaluated by Permutational multivariate analysis of variance (PERMANOVA) statistical tests using the Adonis function (R package “*vegan*”). The effects of bacterial wilt disease in single rhizocompartments were also assessed by PERMANOVA. One-way analysis of variance (ANOVA) followed by Tukey’s test was used to determine the statistical significance among groups. Venn diagram and heatmap analyses were performed using the “*venndiagram*” and “*pheatmap*” packages in R. The Ternary plots were visualized by calculating the mean relative abundances of ASVs of each compartment using the “*vcd*” package in R. The SourceTracker model was applied to predict the relative contributions of different microbial compartments in the enrichment process from bulk soil communities to root endosphere communities. The percentage values were calculated with the functions “*sourcetracker*” and “*predict*” packages in R (Knights et al., 2011).

### Random matrix theory based molecular ecology networks

Molecular ecological networks for root-associated microbiomes under both healthy and diseased conditions were constructed *via* the online pipeline (http://ieg2.ou.edu/MENA/). For each network, ASVs (relative abundance > 0.01%)) presented in at least 4 out of the 6 biological replicates were included for network analysis. The value of *St* was automatically determined by a random matrix theory (RMT) based approach that observed the transition point of nearest-neighbor spacing distribution of eigenvalues from Gaussian to Poisson distribution (Zhou et al., 2011). The networks were visualized using the interactive platforms Gephi and Cytoscape. Nodes represent the individual ASVs, and edges represent the pairwise correlations between the nodes in the microbiome network, indicating biologically or biochemically meaningful interactions. Node topologies could be classified into four categories based on the value of within-module connectivity (*Zi*) and among-module connectivity (*Pi*): peripheral nodes (*Zi* ≤ 2.5 and *Pi* ≤ 0.62), connectors (*Zi* ≤ 2.5 and *Pi* > 0.62), module hubs (*Zi* > 2.5 and *Pi* ≤ 0.62), and network hubs (*Zi* > 2.5 and *Pi* > 0.62) (Olesen et al., 2007).

## Results

### Both niches and disease affect the distribution and source of root-associated microbiomes

**Figure 1:**
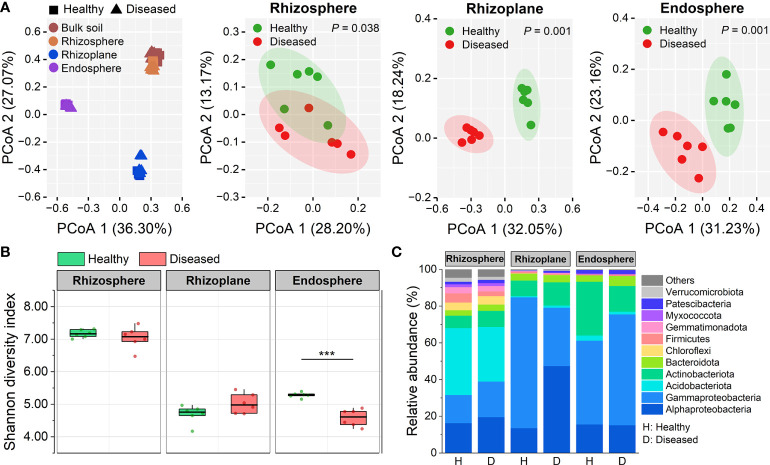
Community assembly of root-associated microbiomes. **(A)** Principal coordinate analysis (PCoA) of Bray–Cutis dissimilarity matrices showing effects of compartment niche and bacterial wilt disease on the community structure of the rhizosphere, rhizoplane and endosphere microbiomes. **(B)** Shannon diversity index of bacterial communities in root-associated microbiomes of healthy and diseased plants Asterisks denote significant differences (***P < 0.001). **(C)** Stacked bar chart showing phylum composition in the three rhizocompartments of healthy and diseased plants based on relative abundance data.

A total of 2,800,052 bacterial 16S rRNA high-quality sequences were obtained from 48 samples (average: 58,334; range: 44,904–75,018 reads per sample). After removing the chimeric and organelle sequences with USEARCH, 14,416 ASVs were identified from these reads. The results showed that the root-associated microbiomes have different patterns of community structure and microbial diversity among the rhizosphere, rhizoplane, and root endosphere compartments (**Figures 1**, **S2**, **S3**). PCoA of Bray–Curtis distance revealed that the bulk soil and root-associated microbiomes formed three distinct clusters regardless of plant health conditions (**Figure 1A**). PERMANOVA analysis was performed to evaluate the relative contribution of rhizocompartment niche and bacterial wilt disease to the structures of root-associated microbiomes. The calculation results indicated that compartment niche explained a large source of variation within the microbiome data (63.54%, *P* < 0.001), and bacterial wilt disease showed a relatively little effect on the differences of root-associated microbiomes (6.85%, *P* < 0.001). PCoA of each compartment microbiome showed that bacterial wilt disease caused significant differences in the community structures of the rhizosphere, rhizoplane, or root endosphere (PERMANOVA; *P* < 0.001) (**Figure 1A**), but the structure of the bulk soil community was not significantly affected by the disease (PERMANOVA; *P* = 0.105) (**Figure S1**).

Measures of the alpha diversity (Shannon index, Chao1 richness, and Simpson index) showed an obvious downtrend from the rhizosphere to the endosphere (**Figures 1B**, **S2**). The bacterial alpha diversity in the rhizosphere and rhizoplane compartments was not affected by bacterial wilt disease (ANOVA; *P* > 0.05). However, the alpha diversity in the diseased endosphere was significantly lower than those in the healthy one in terms of Shannon diversity index and Simpson index (ANOVA; *P* < 0.001) (**Figures 1B**, **S2**). Taxonomic classification showed that both bacterial phyla and genera varied distinctly across compartment niches (**Figures 1C**, **S3**). The rhizoplane and endosphere had a significantly greater proportion of Gammaproteobacteria, Actinobacteria, and Bacteroidota than the rhizosphere, whereas Acidobacteria, Chloroflexi, Firmicutes, Gemmatimonadota, and Verrucomicrobiota were mostly depleted in the rhizoplane and endosphere compared with the rhizosphere (**Figure 1C**). The composition differences among the three rhizocompartments differed more significantly at the genus level (**Figure S3**). The three compartments shared only 138 genera and the rhizosphere had the largest number of distinct genera (165), followed by the rhizoplane (110). Besides, the taxonomic classification of each compartment showed that different compartments changed distinct members under bacterial wilt disease, and the rhizoplane and endosphere were more affected by the disease than the rhizosphere (**Figures 1C**, **S3**). For example, *Allorhizobium-Neorhizobium-Pararhizobium-Rhizobium*, *Ensifer*, and *Sphingomonas* were significantly enriched in diseased rhizoplane, while *Pseudomonas* and *Stenotrophomonas* were significantly enriched in diseased endosphere.

**Figure 2:**
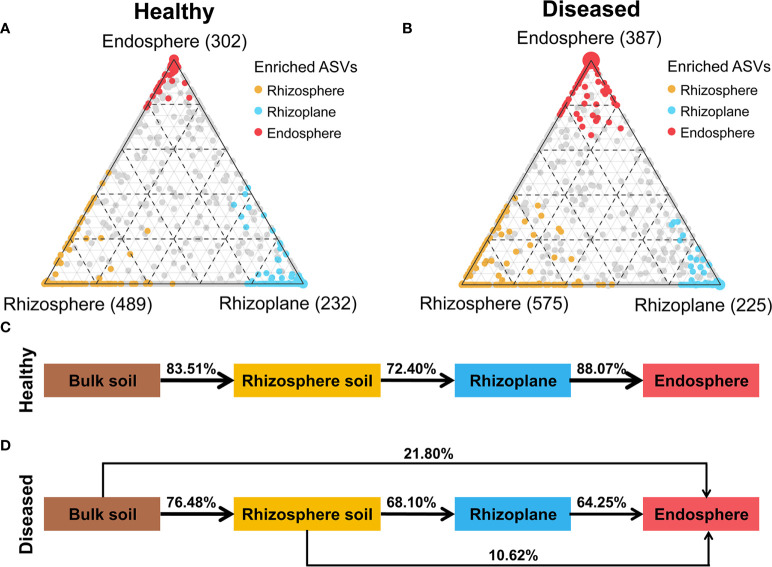
Distribution of ASVs across different compartments. **(A, B)** Ternary plots depicting bacterial ASVs significantly enriched in three root-associated microbiomes (FDR, *p* < 0.01) under healthy and diseased conditions. Each circle represents one ASV, and the size of each circle represents its relative abundance. Different colors represent different compartments. **(C)** Source-tracking analysis showing the sources of the rhizosphere, rhizoplane and endosphere microbiomes in healthy plants. **(D)** Source-tracking analysis showing the sources of the rhizosphere, rhizoplane and endosphere microbiomes in diseased plants.

To understand better how the bacterial wilt disease influenced the bacterial community, the specifically enriched ASVs in each compartment and the microbial sources of each compartment across healthy and diseased samples were compared (**Figure 2**). Ternary plots showed that more specific ASVs were enriched in the rhizosphere than the rhizoplane and endosphere under both healthy and diseased conditions. Compared with healthy samples (489 and 302 enriched ASVs in the rhizosphere and endosphere, respectively), there were significantly more ASVs were enriched in the diseased rhizosphere (579) and endosphere (387), while the specific ASVs in rhizoplane showed a slight decrease (from 232 to 225) (**Figures 2A**, **B**). SourceTracker analysis showed that root-associated microorganisms were mainly derived from the bulk soil, but the trends differed in healthy and diseased samples (**Figures 2C**, **D**). In healthy samples, the majority of the rhizosphere microbial members (83.51%) were derived from the bulk soil, then the microorganisms were gradually filtered at the rhizoplane and endodphere compartments. Most of the endosphere members (88.07%) were migrated from the rhizoplane and no microorganisms were from the rhizosphere or bulk soil (**Figure 2C**). In diseased samples, the rhizosphere microorganisms (76.48%) were also primarily derived from the bulk soil and gradually filtered at different plant compartment niches. However, for the root endosphere microorganisms in diseased samples, the bulk soil (21.80%) and the rhizosphere (10.62%) were also important sources of the endosphere microbiome except for the rhizoplane (64.25%) (**Figure 2D**).

All the above results showed that pathogen invasion could affect the distribution of root-associated microbiomes and promote the plant root system to expand the ecological range of recruiting microorganisms.

### Three Rhizo-compartments enriched significantly different OTUs under disease

**Figure 3:**
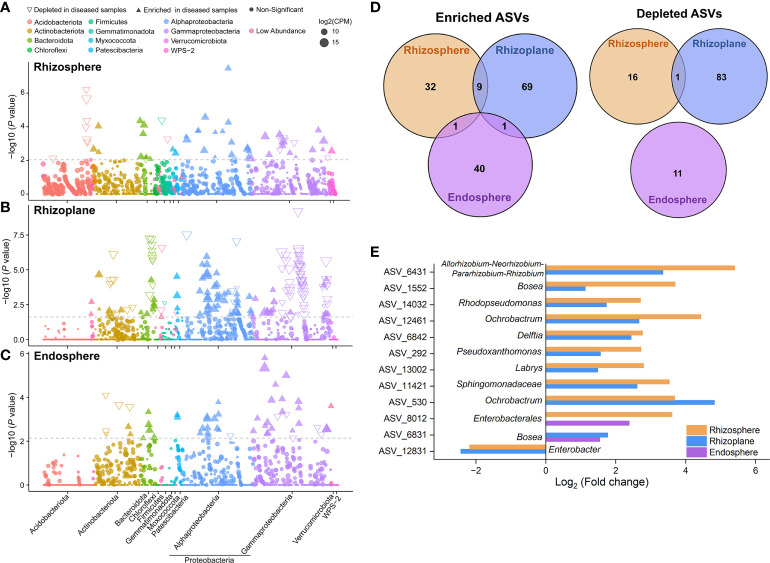
Taxonomic characteristics of differential bacteria of root-associated microbiomes between healthy and diseased plants. **(A–C)** Manhattan plots showing ASVs enriched or depleted in the diseased rhizosphere, rhizoplane, and endosphere, respectively. Each circle or triangle represents a single ASV. ASVs enriched or depleted in the diseased samples are represented by filled or empty triangles, respectively (ASVs abundance > 0.01%, *P* < 0.05). **(D)** Venn diagram depicting number of enriched or depleted ASVs in each compartment of diseased plants. **(E)** The genus classification (if not annotated to specific genera, using higher-level taxa to represent) of the shared enriched or depleted ASVs between the microbiomes of diseased rhizosphere, rhizoplane, and endosphere.

Given the recruitment strategy of plants for beneficial microbes, we identified enriched or depleted ASVs (relative abundance > 0.01%) in each rhizocompartment of diseased samples using Manhattan plots (**Figure 3**). In the three compartments, ASVs enriched in diseased samples belonged to a wide range of bacterial phyla, including Actinobacteriota, Bacteroidta, Patescibacteria, Proteobacteria (mainly Alphaproteobacteria and Gammaproteobacteria), and WPS-2 (FDR adjusted *P* < 0.05, Wilcoxon rank sum test; **Figures 3A–C**). The rhizosphere and endosphere of diseased samples enriched the same number of ASVs (42). Among them, *Dyadobacter* ASV_6201, *Ochrobactrum* ASV_530 and *Pseudomonas* ASV_4114 in the rhizosphere and *Shinella* ASV_8061 in the endosphere were the “new microbes” in diseased samples, which were present in ≥ 50% diseased samples and in none healthy samples (**Figures 3A, C** and **Tables S2**, **S4**). In comparison, the diseased rhizoplane enriched for more ASVs (mainly belonging to Alphaproteobacteria) while simultaneously depleting a larger proportion of ASVs (79 vs. 84) (**Figure 3D**). Moreover, the rhizoplane of diseased samples presented a large number of distinct ASVs (new microbes) unique to healthy samples, which was distinctly different from the endosphere community (**Figure S4**). Among the enriched ASVs of the rhizoplane, *Mycobacterium* ASV_11679 and *Saccharimonadales* ASV_12660 represented “new microbes” in diseased samples (**Figure 3B** and **Table S3**). In terms of enriched ASVs, none was shared by the three compartments, and only 11 ASVs were shared by two of them (**Figure 3D**). The shared ASVs were assigned to different genera, such as *Allorhizobium-Neorhizobium-Pararhizobium-Rhizobium*, *Bosea*, *Rhodopseudomonas*, *Ochrobactrum*, *Pseudoxanthomonas*, *Labrys* and so on (**Figure 3E**). For depleted ASVs, only one ASV belonging to *Enterobacte*r was shared by the rhizosphere and rhizoplane. The above results indicated that different ASVs were enriched in different compartments with pathogen invasion.

### Molecular ecological networks of root-associated microbiomes are influenced by disease

**Figure 4:**
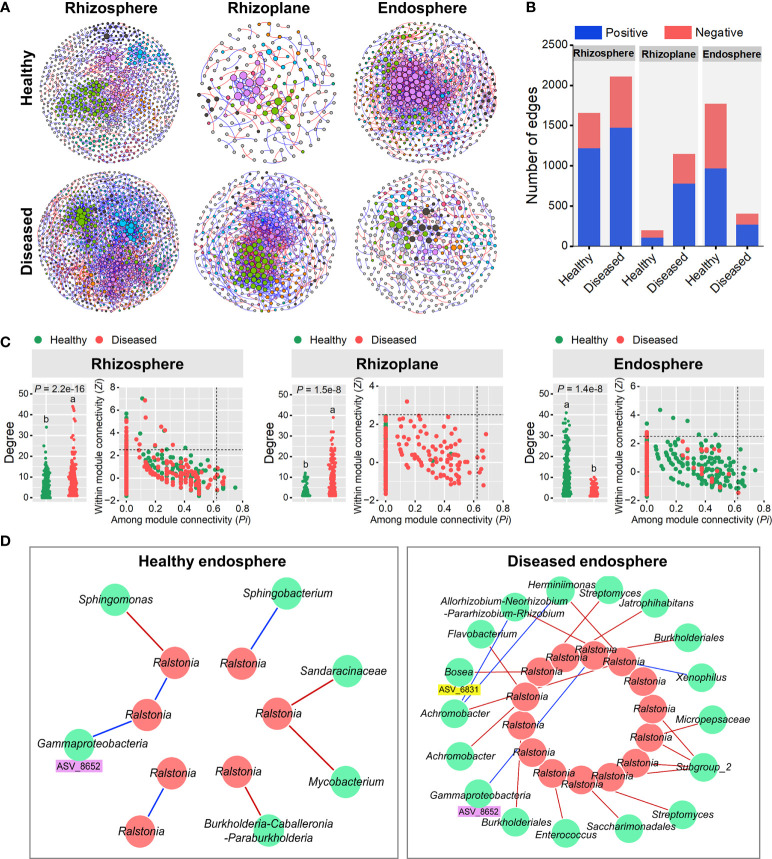
Random matrix theory (RMT)-based molecular ecology networks. **(A)** Bacterial co-occurrence networks of the rhizosphere, rhizoplane and endosphere in healthy and diseased plants. Each node represents an ASV. The size of each node is proportional to the number of connections (that is, degree) and the colors of nodes represent different modules. The links between the nodes indicate strong and significant (*P* < 0.01) correlations. A red line indicates a positive interaction between two individual nodes, while a blue line indicates a negative interaction. **(B)** The number and types (positive or negative) of edges in the rhizosphere, rhizoplane and endosphere networks of healthy and diseased plants. **(C)** Node degree and classification for each root-associated microbiome of healthy and diseased plants. Different letters indicate a significant difference determined by ANOVA test. Each symbol represents an ASV. The topological role of each ASV was determined according to the scatter plot of within-module connectivity (*Zi*) and among-module connectivity (*Pi*). Module hubs have *Zi*> 2.5, whereas connectors have *Pi* > 0.62. **(D)** Subnetworks of the pathogen (*Ralstonia*) and related bacterial species in the root endosphere microbiomes of healthy and diseased plants. Each node represents an ASV. A red line indicates a positive interaction between two individual nodes, while a blue line indicates a negative interaction.

Molecular ecological networks (MENs) were constructed to unravel how bacterial wilt disease affected the microbial interactions across the three rhizocompartments under healthy and diseased conditions (**Figure 4**). The topological properties of the six networks were shown in **Supplementary Table S5**. The same threshold values were chosen for each compartment (0.94 for the rhizosphere, 0.93 for the rhizoplane and 0.94 for the endosphere). The node and link numbers of the rhizosphere networks were significantly higher than those of the rhizoplane and endosphere networks (**Table S5**). For healthy plants, the networks of the rhizosphere (1659 links) and endosphere (1774 links) were more complex than the rhizoplane network (198 links). However, under diseased conditions, the links within networks showed a clear downward trend from the rhizosphere (2110 links) to endosphere (406 links), and the network of the rhizoplane presented a higher number of connections per node (average degree = 5.892) than that of the rhizosphere (3.764) and endosphere (2.115), indicating a highly connected microbial community of the diseased rhizoplane (**Figures 4A, B** and **Table S5**). For different compartments, networks of the rhizoplane and endosphere were significantly affected by the disease (based on the average degree) (**Figure 4C**). In addition, we identified the keystone nodes (connectors and module hubs) in each network based on the values of within-module connectivity (*Zi*) and among-module connectivity (*Pi*), and found 24 connectors (healthy: 9, diseased:15) and 34 module hubs (healthy: 18, diseased: 16) in the rhizosphere networks, 8 connectors (diseased: 8) and 1 module hub (diseased: 1) in the rhizoplane networks, and 14 connectors (healthy: 13, diseased: 1) and 10 module hubs (healthy: 7, diseased: 3) in the endosphere networks (**Figure 4C** and **Tables S6**–**S8**).

Considering the high abundance of *Ralstonia* in the root endosphere (**Figure S3**), we further constructed the endosphere subnetworks of the interactions among pathogenic *Ralstonia* and other microbial members to identify key organisms (**Figure 4D**). The results showed that more bacterial members directly connected to *Ralstonia* in the diseased root than those in the healthy one. For the diseased endosphere, most of the bacterial members relating to *Ralstonia* tended to co-exclude (negative correlations, red lines) rather than co-occur (positive correlations, blue lines) (**Figure 4D**). A variety of bacterial genera such as *Allorhizobium-Neorhizobium-Pararhizobium-Rhizobium, Streptomyces, Burkholderiales, Achromobacter, Bosea and Flavobacterium* were negatively correlated with the pathogenic *Ralstonia* in the diseased root endophytic communities. Among these nodes, *Bosea* ASV_6831 was significantly enriched (FDR adjusted *P* < 0.05) in diseased root endosphere, which provided evidence that plant recruited some microorganisms to suppress the expansion of pathogenic *Ralstonia*. Only two positive correlations between bacterial genera (*Xenophilus* and an unclassified genus of Gammaproteobacteria) and the pathogen were found in the root endosphere. One node (ASV_8652) belonging to *Gammaproteobacteria* and positively correlating to *Ralstonia* was detected in both the healthy and diseased networks of the endosphere, indicating its importance in facilitating the colonization and development of the pathogen (**Figure 4D**).

Collectively, molecular ecological networks of each compartments were influenced by bacterial wilt disease, but the variation trends were significantly between them.

### The function profiles of root-associated microbiomes are influenced by disease

**Figure 5:**
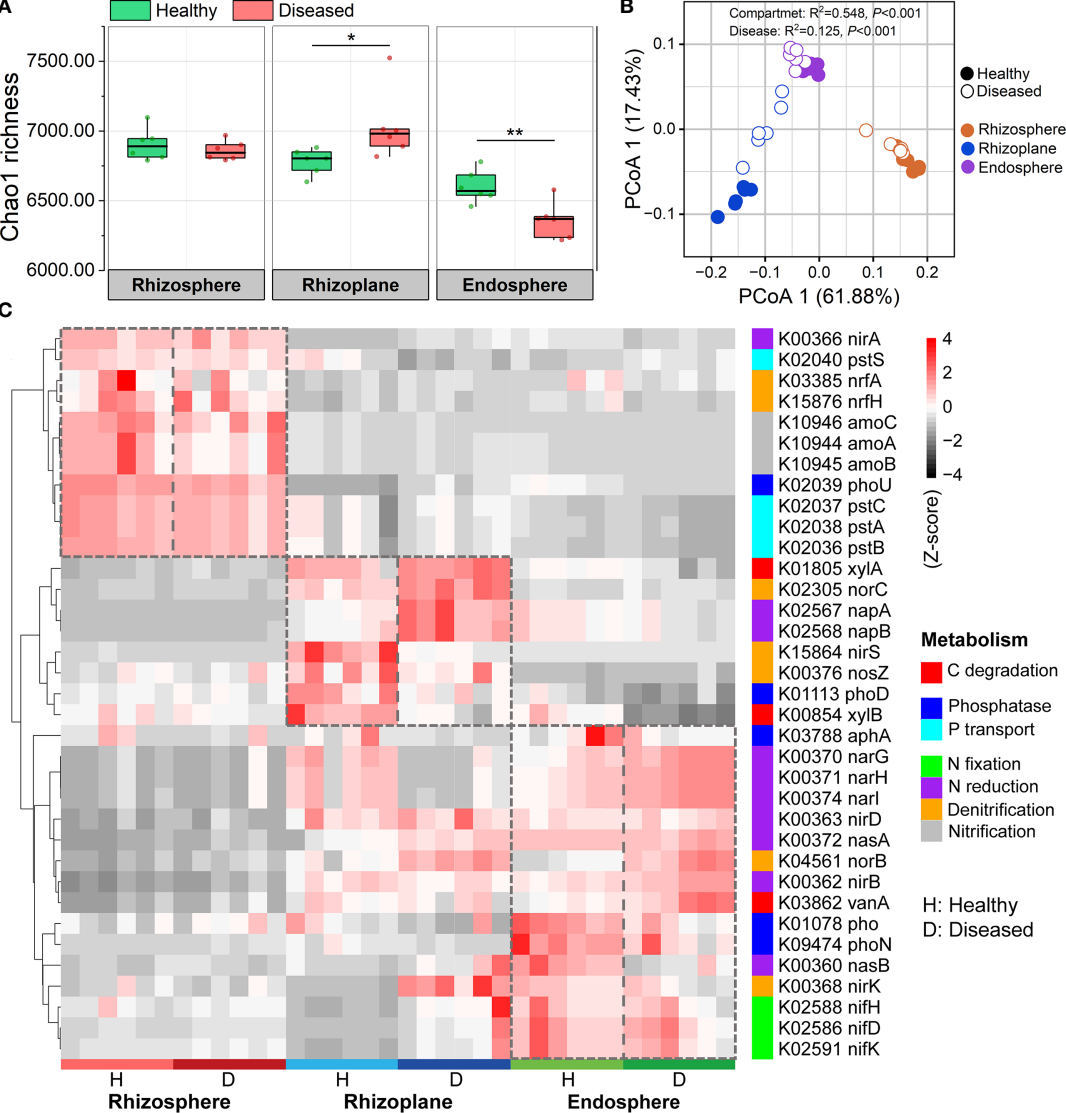
PICRUSt predicted metagenome functions at KO level. **(A)** Functional diversity of KO profiles of the rhizosphere, rhizoplane and endosphere microbiomes in healthy and diseased plants. Asterisks denote significant differences (*P < 0.05; **P < 0.01). **(B)** PCoA analysis of functional genes based on Bray–Curtis distance matrices of KO. **(C)** Heatmap exhibiting the relative abundance of functional genes (based on KO) involved in C, N, and P cycling which varied among three root-associated microbiomes.

In order to investigate the effects of disease on the community functions of different compartments, metagenomes of bacterial communities were predicted using PICRUSt2 and then annotated by referring to the KEGG database. A total of 7,986 KOs (KEGG Orthologs) were predicted in the three root-associated communities. Our results showed that the endosphere microbiome in the diseased roots possessed significantly lower KO diversity (i.e. Chao1 richness) than those in the healthy roots (ANOVA; *P* < 0.01). However, the diseased rhizoplane microbiome showed a higher KO diversity than the healthy one (ANOVA; *P* < 0.05) (**Figure 5A**). PCoA analysis at the KO level showed that community functions of the three compartments significantly differed from each other (R^2^ = 0.548, *P* < 0.001), and the bacterial wilt disease also had a significant effect on microbiome functions for the three compartments (R^2^ = 0.125, *P* < 0.001) (**Figure 5B**). Several C, N and P cycling related genes showed a varied pattern among three root-associated communities (**Figure 5C**). Specifically, functional genes involved in Nitrification (e.g. *amo*A, *amo*B, and *amo*C) and P transport (e.g. *pst*A, *pst*B *pst*C, and *pst*S) were more abundant in the rhizosphere microbiome, genes related to C degradation (e.g. *xyl*A and *xyl*B) and Denitrification (e.g. *nor*C, *nir*S, and *nos*Z) were more abundant in the rhizoplane microbiome, and N reduction genes (e.g. *nar*G, *nar*H, *nar*I, *nas*A, *nas*B, and *nir*D) and N fixation genes (e.g. *nif*H, *nif*D, and *nif*K) were more abundant in the endosphere microbiome (**Figure 5C**). For individual compartment, disease presented more significant effects on the rhizoplane and endosphere than those on the rhizosphere. For the rhizoplane and endosphere microbiomes, the genes involved in N reduction were enriched and P transport genes were depleted in diseased samples (**Figure 5C**).

**Figure 6:**
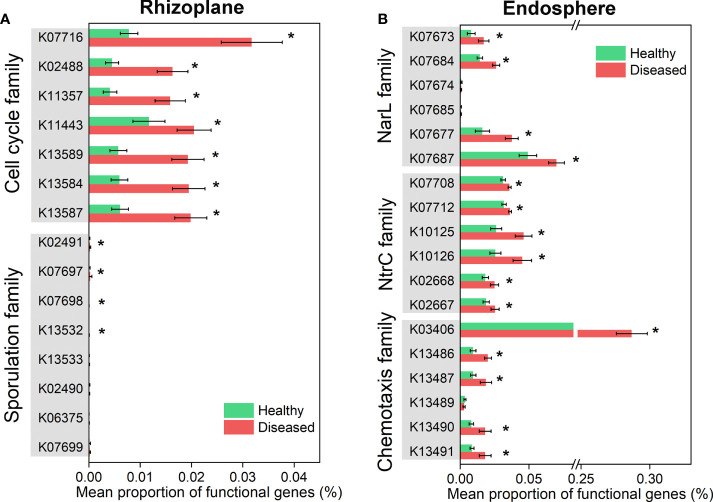
Differential abundance analysis of microbiome functional genes involved in plant-microbiome signaling pathways which varied significantly in healthy and diseased rhizoplane or endosphere. **(A)** Functional genes in cell cycle and sporulation families showing significantly different between healthy and diseased rhizoplane microbiomes. **(B)** Functional genes in NarL, NtrC and chemotaxis families showing significantly different between healthy and diseased endosphere microbiomes. Asterisks denote significant differences (*P < 0.05).

We further compared the functional genes involved in plant-microbiome signaling pathways (e.g. signal transduction) (**Figure S5**, **Figure 6**). The distributions of signal transduction genes were obviously different across the three root-associated microbiomes, and the microbiomes of the rhizoplane and endosphere were more susceptible to disease (**Figure S5**). For instance, the relative abundance of genes associated with cell cycle family and sporulation family was significantly increased in the diseased rhizoplane microbiome (ANOVA; *P* < 0.05), compared with the healthy plant (**Figure 6A**). The relative abundance of the functional genes involved in chemotaxis family (e.g. K03406: methyl-accepting chemotaxis), NtrC family and NarL family was significantly abundant in the microbiome of the diseased root endosphere (ANOVA; *P* < 0.05), compared with the healthy plant (**Figure 6B**).

All the above results indicated that the community function of endosphere and rhizoplane were significantly changed with pathogen invasion, but.

## Discussion

Uncovering the changes of the structure and assembly of plant-associated microbiomes with plant disease invasion is essential to advance the co-evolutionary theory of plant-microbiome interactions (Liu et al., 2020). In this study, we characterized the compositions of three distinct rhizocompartments—the rhizosphere, rhizoplane, and endosphere, and gained insights into the effects of plant disease on each of these compartments. Our results demonstrated that the community assembly of the three root-associated microbiomes was simultaneously influenced by compartment niche and bacterial wilt disease. The microbiomes in or close to the plant’s root (endosphere and rhizoplane) were more sensitive to the plant disease than the rhizosphere microbiome in terms of multiple microbial attributes (i.e. alpha-diversity, community structure, microbial networks, and community functions). However, the response strategies of the endosphere and rhizoplane microbiomes were obviously different.

Previous studies have demonstrated that the plant compartment was a crucial factor affecting the assembly of plant-associated microbiomes (Cregger et al., 2018; Gao et al., 2021). Similar to these reports, our results showed that the three rhizocompartments formed distinct microbial communities regardless of plant health conditions (**Figure 1A**). Microbial diversity (Shannon index, Chao1 richness, and Simpson index) patterns showed an obvious gradient from the rhizosphere to the endosphere (**Figure 1B**), indicating the filter effect of plant hosts on the closely associated microbiome assembly (Reinhold-Hurek et al., 2015). We noted that the proportion of Proteobacteria is more abundant in the rhizoplane and endosphere compartments compared with the rhizosphere and that the relative abundances of Acidobacteria and Gemmatimonadota were higher in the rhizosphere (**Figure 1C**), which were similar to the microbial composition of other plant species, such as *Arabidopsis* (Schlaeppi et al., 2014), rice (Edwards et al., 2015) and citrus (Zhang et al., 2017). Therefore, we inferred that the distribution of different bacterial phyla associated with the plant roots might be similar for most land plants. Further, functional analysis in our study revealed that the functional diversity and enriched functional traits in microbial communities varied across the three rhizocompartments (**Figure 5**). We demonstrated that the genes involved in nitrate reduction (*nar*G, *nar*H, and *nar*I) and N assimilation gene (*nas*A and *nas*B) were enriched from the rhizosphere to the endosphere (**Figure 5C**), further suggesting the importance of nitrogen resources supply of plants for rhizo-microbiomes.

We found that bacterial wilt disease had different effects on communities of the three rhizocompartments, in which the endosphere was more sensitive to the disease (**Figure 1**). Generally, the low microbial diversity of the microbial community was beneficial for the pathogen invasion (Locey and Lennon, 2016). Indeed, in our study, the endosphere, which held the lowest diversity indexes of Shannon, Chao1, and Simpson (**Figure 1B**), showed a significantly higher abundance of the pathogen (*Ralstonia*) (**Figure S3**). With the success of pathogen invasion, the endosphere microbiome was disrupted and the community structure and function were clearly altered (Wei et al., 2017). Previous studies have demonstrated that the root-associated bacterial assemblage is mostly derived from the highly diverse soil microbiome surrounding roots (Zgadzaj et al., 2016). Here, we found the bacterial source of the endosphere community was also influenced by the pathogen invasion. In healthy plants, rare microorganisms were observed from the rhizosphere or bulk soil to the endosphere, while the bulk soil (21.80%) and the rhizosphere (10.62%) became important sources of the endosphere microbiome in diseased samples (**Figure 2C, D**). The results were in accordance with a recent study that endophytic communities of the infected plants were also derived from soil communities (Hu et al., 2020). The pathogenic *Ralstonia* enriched in the endosphere may begin in the soils and transfer to the plant root.

Faced with complex environments, plants may actively seek help from microbes to relieve stresses. Previous studies have evidenced that plants with pathogen invasion could use a variety of chemical stimuli to recruit beneficial microbes/traits from the environment (Liu et al., 2021). The beneficial microbes attracted by plants acted as key members of plant microbiomes and enhanced plant resistance to disease by interacting directly with pathogens (e.g., competition and antibiosis) (Woo et al., 2006) or by modulating plant defense responses (Mejía et al., 2014). For example, plant roots of sugar beet enriched for Chitinophagaceae and Flavobacteriaceae in the root endosphere, and the reconstruction of a consortium of *Chitinophaga* and *Flavobacterium* consistently suppressed fungal root disease (Carrión et al., 2019). Here, the enriched bacterial members across three root-associated microbiomes were significantly different (**Figure 3**), indicating microbial colonization in plant-associated niches is not a passive process and that plants had the ability to select for certain microbial members or that different microbes tended to enrich in the favorable colonizing niches (Edwards et al., 2015). Among these enriched microbes, most of them were enriched from the native community and distributed in a variety of bacterial genera. Besides, in the diseased plants, some new microbes (at ASV level) appeared in each rhizocompartment (**Figures 3** and **Tables S2**–**S4**). For example, compared to the healthy samples, *Dyadobacter* ASV_6201, *Ochrobactrum* ASV_530, and *Pseudomonas* ASV_4114 in the rhizosphere, *Mycobacterium* ASV_11679 and *Saccharimonadales* ASV_12660 in the rhizoplane and *Shinella* ASV_8061 in the endosphere represented the “new microbes” in the three diseased compartments. These new ASVs may carry new microbial functions to help plants to resist the invasion of pathogenic bacteria. For example, *Pantoea* asv90 and *Methylobacterium* asv41 were identified as “recruited new microbes” in citrus leaves and exhibited antagonistic activities to the melanose pathogen *Diaporthe citri* (Li et al., 2022).

Except for analyzing changes in microbial taxa, changes in microbial interactions and functional profiles can further provide useful information to the effects of microbiome changes on plant health. Our study here revealed that networks of the rhizoplane and endosphere were significantly affected by the disease (based on links and the average degree) (**Figure 4**), which may be related to the low bacterial diversity in the rhizoplane and endosphere microbiomes (**Figure 1B**). However, the variation patterns of the rhizoplane and endosphere networks were greatly different. The rhizoplane microbiome of diseased samples presented a more intense microbial network than that of healthy samples, but the endosphere microbiome showed exactly the opposite variation. It has been suggested that highly connected networks could occur when microbes faced environmental pressures, such as pathogen invasion (Faust and Raes, 2012). The complex soil microbial community networks, rather than the simple ones, benefit plants (Yang et al., 2017). Therefore, the complex networks observed in the diseased rhizoplane might be a beneficial signal for plant defense. Although the endosphere network of diseased samples was simple as a whole, more microbes negatively connected to *Ralstonia* were identified in the diseased root than those in the healthy root (**Figure 4D**). The microbes having positive or negative connections with pathogen were considered as “pathogen antagonists” or “pathogen facilitators”, respectively (Tao et al., 2021). Therefore, the microbes belonging to the genera of *Allorhizobium-Neorhizobium-Pararhizobium-Rhizobium*, *Streptomyces, Burkholderiales, Achromobacter, Bosea, and Flavobacterium* might act as ‘pathogen antagonists’ (**Figure 4D**). Notably, *Bosea* ASV_6831, a potential ‘pathogen antagonist’, was significantly enriched (FDR adjusted *P* < 0.05) in diseased root endosphere, might play a crucial role in suppressing the growth of pathogenic *Ralstonia*. *Bosea* spp. are Gram-negative, rod-shaped aerobic bacteria and are frequently isolated from various environments, including soils, sediments, root nodules of plants and digester sludge (Ouattara et al., 2003; De Meyer and Willems, 2012; Safronova et al., 2015). Several studies have reported that *Bosea* could protect plants against phytopathogen fungi, such as *Botrytis cinerea* and *Penicillium expansum* (Khoury et al., 2021). Here, our results indicated that *Bosea* ASV_6831 might also be a potential new ‘pathogen antagonist’ to phytopathogen bacteria, in accordance with a recent study that *Bosea minatitlanensis* SSB-9 had plant growth-promoting and antibacterial activities against *Ralstonia solanacearum* (Yuan et al., 2022). At present, there are few studies on the antagonistic mechanism of *Bosea* against plant pathogens, and future studies should focus on the potential interactions between *Bosea* and pathogens.

Analysis of community function indicated that microbiome functional genes involved in sporulation family and cell cycle family were enriched in the diseased rhizoplane and those involved in chemotaxis family, NtrC family, and NarL family were more abundant in the diseased endosphere when compared to those genes in healthy samples. With the pathogen invasion, microbes in the rhizoplane community might take measures to increase their growth rate and promote sporulation formation to enhance their competitiveness with the pathogen. It has become evident that the production of bacterial toxins was controlled by the pathway regulating sporulation initiation (Bongiorni et al., 2007). The toxins produced by microbes could inhibit the growth of pathogens to a certain extent. The increase of genes in NarL and NtrC families in the diseased root endosphere indicated that nitrogen availability might be an important limiting factor for the plant under pathogenic *Ralstonia* invasion. Among the functional genes involved in chemotaxis, the gene (K03406) encoding MCPs (methyl-accepting chemotaxis proteins) was significantly enriched in the microbiome of diseased root endosphere, which was in accordance with a recent study on the Fusarium wilt disease of chili pepper (Gao et al., 2021). Under pathogen invasion, plants can recruit distant beneficial microbes by actively releasing some specific secondary metabolites (Schulz-Bohm et al., 2018; Yuan et al., 2018), which might cause the enrichment of microbial MCP genes in diseased plants. Some bacteria could use MCPs to detect specific concentrations of plant metabolic compounds in the extracellular matrix, enabling the directional accumulation of the bacteria to the plant (Gao et al., 2021).

## Data availability statement

The data presented in the study are deposited in the Genome Sequence Archive in Beijing Institute of Genomics repository (https://bigd.big.ac.cn/gsa), accession number CRA007832.

## Author contributions

PC, JT and JJ designed the experiments. JT, SY, ZY and QC performed the experiments. PL, JJ, YX and ZL analyzed the data. JT and SY wrote the manuscript. JJ and PC reviewed the manuscript. All authors contributed to the article and approved the submitted version.

## Funding

This project was funded by China Postdoctoral Science Foundation (Grant/Award number: 2021M693511) and the Scientific and Technological Project of Henan Province (Grant/Award number: 222102110224 and 212102110257).

## Conflict of interest

The authors declare that the research was conducted in the absence of any commercial or financial relationships that could be construed as a potential conflict of interest.

## Publisher’s note

All claims expressed in this article are solely those of the authors and do not necessarily represent those of their affiliated organizations, or those of the publisher, the editors and the reviewers. Any product that may be evaluated in this article, or claim that may be made by its manufacturer, is not guaranteed or endorsed by the publisher.
