# Supplementary material for: The wilt pathogen induces different variations of root-associated microbiomes of plant
Source: Front Plant Sci. 2022 Sep 16;13:1023837. doi: 10.3389/fpls.2022.1023837 (PMC9523445; doi:10.3389/fpls.2022.1023837)
Supplement: Supplementary file 1 [file DataSheet_1.docx]

Supplementary Material

# Supplementary Figures





**Figure S1.** Principal coordinate analysis (PCoA) of Bray–Cutis dissimilarity matrices showing effects of bacterial wilt disease on the composition of bulk soil.


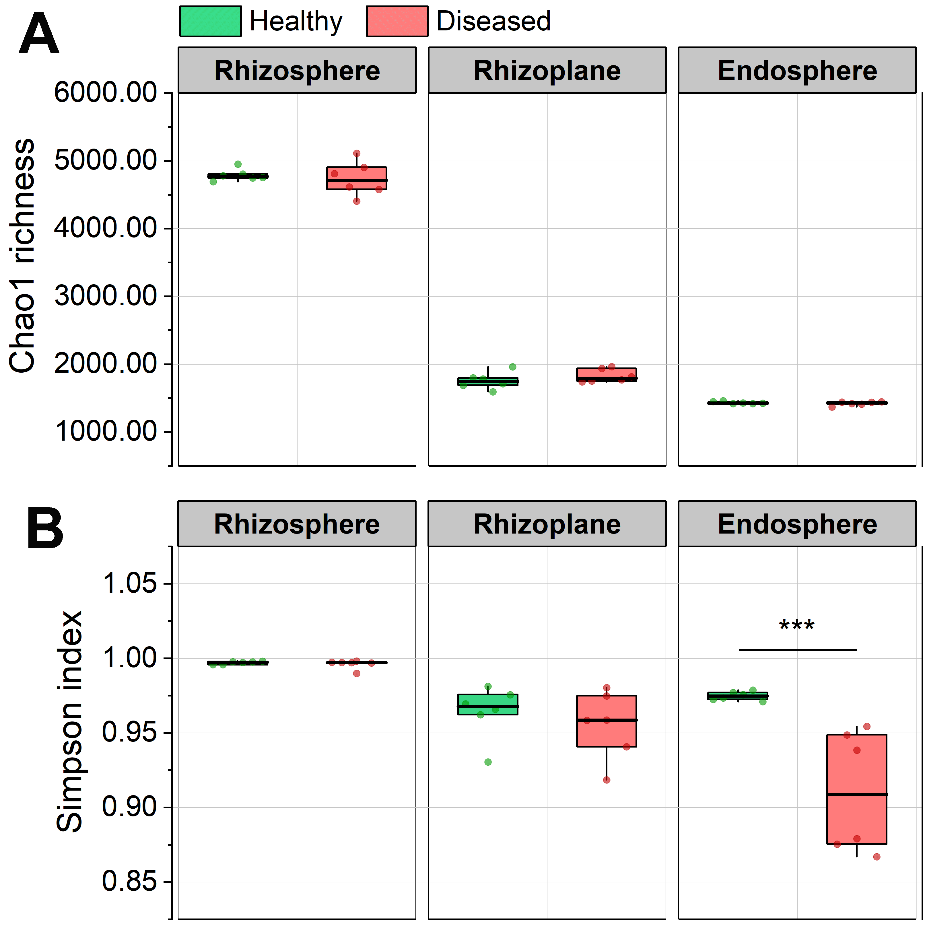


**Figure S2.** Chao1 richness (A) and Simpson index (B) of bacterial communities in root-associated microbiomes (rhizosphere, rhizoplane and root endosphere) of healthy and diseased plants.


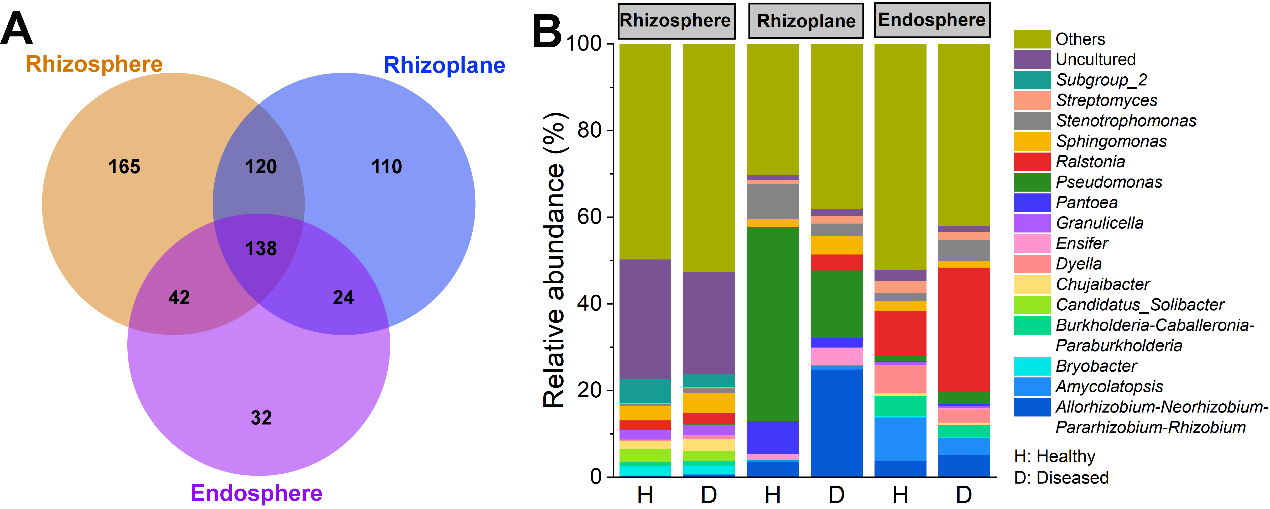


**Figure S3.** The microbial composition of root-associated microbiomes at the genus level. (A) Venn diagram depicting number of genera identified across the rhizosphere, rhizoplane and endosphere microbiomes. (B) Stacked bar chart showing genus composition of the rhizosphere, rhizoplane and endosphere microbiomes based on relative abundance data.


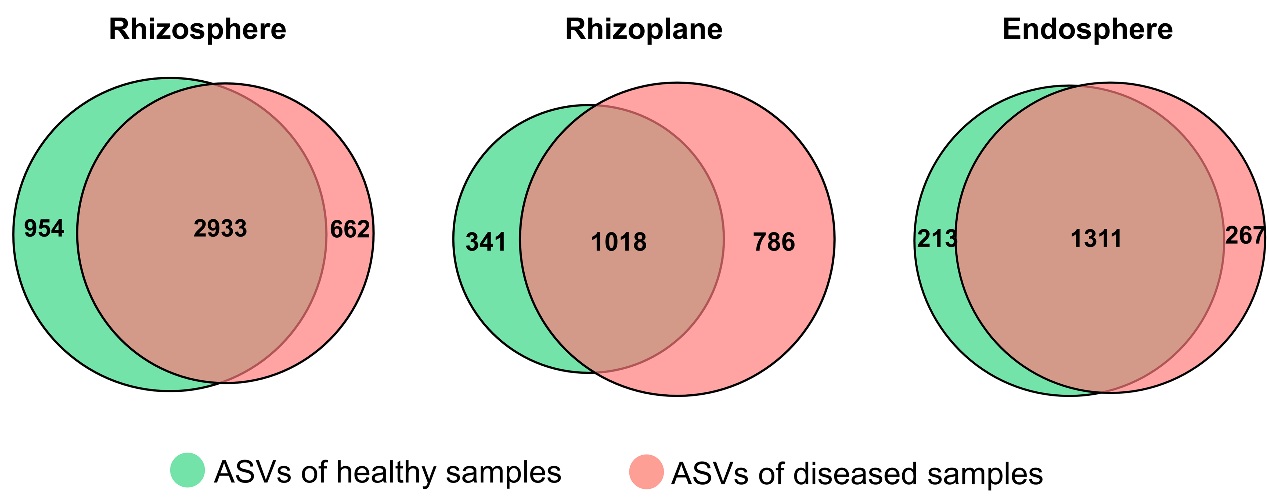


**Figure S4.** Venn diagram depicting the number of ASVs identified in the rhizosphere, rhizoplane and endosphere from healthy and diseased plants.


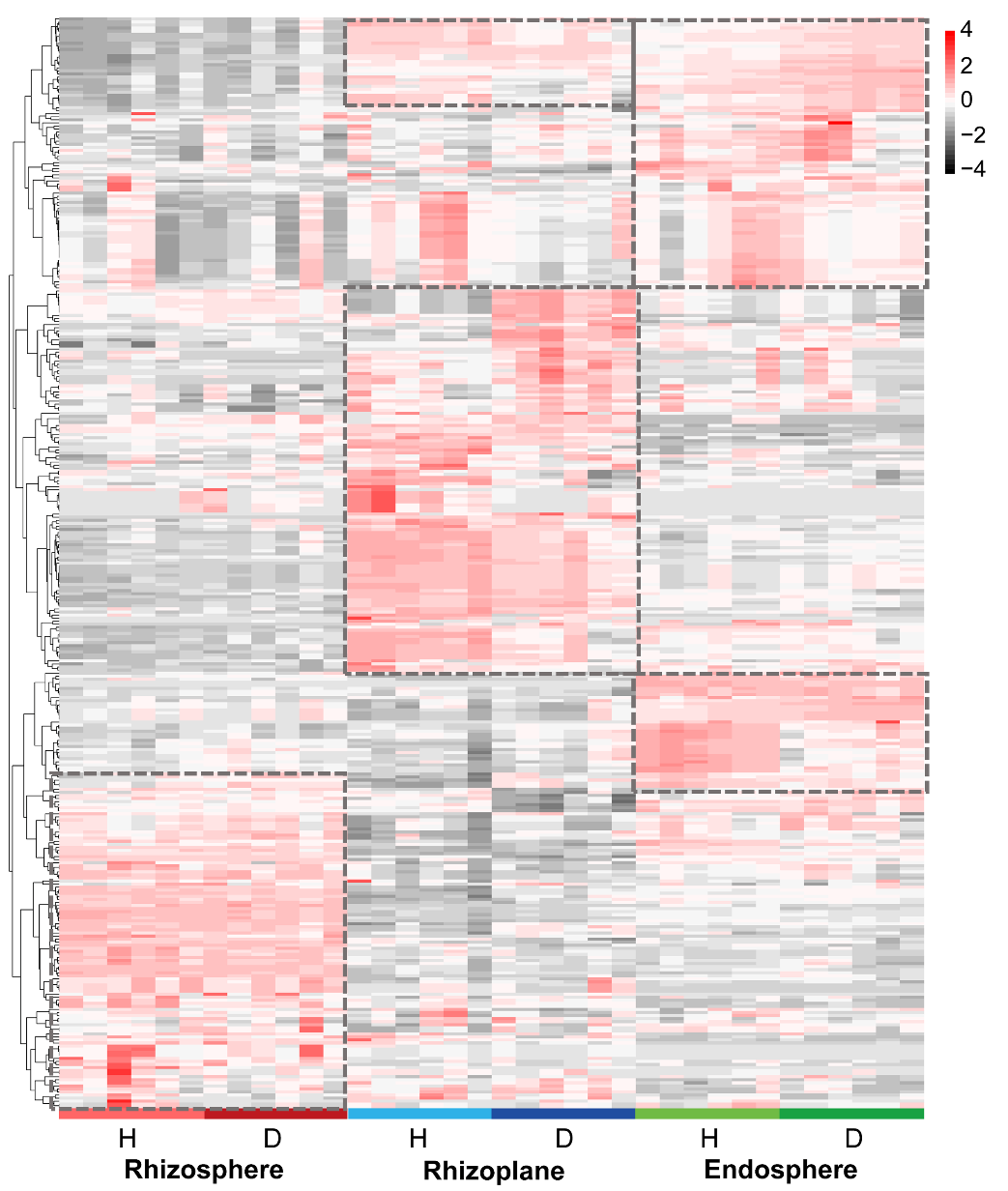


**Figure S5.** Heatmap exhibiting the relative abundance of functional genes (based on KO) involved in plant-microbiome signaling pathways between healthy and diseased plants among three root-associated microbiomes.
